# A New Method for Forensic DNA Analysis of the Blood Meal in Chagas Disease Vectors Demonstrated Using *Triatoma infestans* from Chuquisaca, Bolivia

**DOI:** 10.1371/journal.pone.0003585

**Published:** 2008-10-30

**Authors:** Juan Carlos Pizarro, Lori Stevens

**Affiliations:** 1 Department of Biology, University of Vermont, Burlington, Vermont, United States of America; 2 Facultad de Bioquímica, Universidad de San Francisco Xavier de Chuquisaca, Sucre, Bolivia; University of California Los Angeles, United States of America

## Abstract

**Background:**

Feeding patterns of the vector are important in the epidemiology of Chagas disease, the leading cause of heart disease in Latin America. Chagas disease is caused by the parasite, *Trypanasoma cruzi*, which is transmitted by blood feeding insects. Historically, feeding behaviours of haematophagous insects have been investigated using serological reactions, which have detection limits in terms of both taxonomic resolution, and quantity and quality of the blood meal. They are labor intensive, require technical expertise, need fresh or frozen samples and antibodies often are either not available commercially or the resources for synthesis and purification are not available. We describe an assay to identify vertebrate blood meal sources, and the parasite *T. cruzi* using species-specific PCR assays from insect vectors and use the method to provide information regarding three questions: (1) Do domestic and peri-domestic (chicken coop and animal corral) habitats vary in the blood meals detected in the vectors? (2) What is the pattern of multiple blood meals? (3) Does the rate of *T. cruzi* infection vary among habitats and is it associated with specific blood meal types?

**Methodology/Principal Findings:**

Assays based on the polymerase chain reaction were evaluated for identification of the blood meal source in the heamatophagous Chagas disease vector *Triatoma infestans*. We evaluate a technique to identify 11 potential vertebrate food sources from the complex mixture extracted from the vector's abdomen. We tested the assay on 81 *T. infestans* specimens collected from the Andean highlands in the department of Chuquisaca, located in central Bolivia, one of the regions in South America where sylvatic *T. infestans* have been reported. This area is suggested to be the geographic origin of *T. infestans* and has very high human infection rates that may be related to sylvatic vector populations.

**Conclusion/Significance:**

The results of the assays revealed that a high percentage of insects collected in human dwellings had fed on peri-domestic animals. In contrast, one insect from a chicken coop but no bugs from corrals tested positive for human blood. Forty-eight percent of insects tested positive for more than one vertebrate species. *T. cruzi* infection was detected in 42% of the specimens. From the epidemiological point of view, the results reveal an overall pattern of movement from peri-domestic structures to human habitations for *T. infestans* in this region of Bolivia as well as the important role of pigs, dogs, chickens and guinea pigs in the dynamics of *T. cruzi* infection.

## Introduction

*Triatoma infestans* (Hemiptera:Reduviidae) is the main vector of Chagas disease in seven countries of South America [1]. The vector species is believed to have evolved in and be endemic to the Andean highlands of Bolivia [2]–[4] and its current geographic range includes 55% of Bolivia [5], spanning domestic, peri-domestic and sylvatic habitats [6]–[8]. In 1991 the World Health Organization launched the Southern Cone Initiative targeting vector-borne and blood-transfusion transmission in the “cone” region of South America. This initiative has shown achievement in Argentina, Brazil, Chile and Uruguay as reflected in low rates of house infestation and a decline in human *T. cruzi* infection [9]. Bolivian control activities, started in the mid 1980s, have not been as successful. One possible reason for the disparity is the existence of sylvatic populations of *T. infestans* that disperse from the natural ecotope and colonize recently insecticide treated peri-domestic structures and homes. Additional sources of reinfestation include recrudescence (hatching of eggs laid prior to spraying) [10], [11], survivors of the insecticide treatment [2], [12] and migrants from peri-domestic structures where spraying is likely not as effective. However, regions vary in their success with vector control and local vector ecology may predispose some areas to the persistence of bugs in domestic and peri-domestic habitats. For example, in the southern part of the Bolivian department of Potosí great success was observed in the elimination of *T. infestans* during a one-year trial [5]. However, other endemic zones in Bolivia continue to have active *T. cruzi* transmission and high rates of house infestation despite similar control efforts [13], [14]. Results of studies from Argentina show re-colonizers come from nearby untreated structures in the same homestead, as well as insects dispersing from neighboring localities [15].

Understanding vector movement and feeding preferences are important to the success of insecticide spraying programs against Chagas disease vectors. Infestation of insecticide-treated habitats from recrudescence, colonizers from nearby untreated areas, or sylvatic populations jeopardize the success of control program initiatives. Consequently, the strategy implemented during the surveillance phase of these programs relies on the ability to identify sources of reinvasion. Monitoring methods include investigating the population genetic structure of the vector population and analyzing the spatial association between the capture location of vectors and their feeding sources. Population genetic approaches use a variety of methods including estimates of gene flow among populations based on allozyme variability, [2], [16], [17] and more recently estimates of migration using microsatellites [18], [19].

Historically, feeding patterns of haematophagous insects have been investigated based on serological reactions [20]–[26]. Studies of feeding patters revealed that domestic insects often feed on multiple hosts [24], [25]. Feeding of *Rhodnius prolixus* and *T. dimidiata* collected from houses in Guatemala, examined with ELISA, showed that more had fed on human than other species, both species fed on opossum, and, for the insects examined, only *R. prolixus* fed on chicken and only *T. dimidiata* on cow [24]. In Argentina, dogs, chickens, and humans are the most common blood sources of domestic *T. infestans* [20]. The results of studies of vectors collected from and around houses using a dot-blot assay showed that *Rhodnius pallescenes*, a sylviatic species, collected in houses are more likely to have fed on humans than those collected from palm trees [23]. Insect density and spatial and temporal occurrence of hosts and insects contribute to host choice [20]. The number, identity and proximity of domestic and peri-domestic animals influence feeding patterns [27].

Feeding preference studies have shown that availability can be more important than preference [22]. An experiment examining *T. infestans*, *T. dimidiata*, and *R. prolixus* feeding on opposum, dog, chicken and toad, revealed a preference for homeothermic hosts, *T. infestans* showed a slight preference for dogs and only *T. dimidiata*, the least aggressive species, fed on toads [21]. *T.infestans* from houses in Argentina also showed seasonal preference. Bugs seemed to prefer dogs in the summer and chickens in winter [20].

To understand vector movement, host feeding in different ecotopes has been studied. The number, identity and proximity of domestic and peri-domestic animals influence the dispersal conduct of *T. infestans*. In the peri-domicile the effectiveness of insecticide treatment is reduced due to size, abundant refuge sites and physical conditions that hasten the degradation of residual insecticides [28]. Consequently, in Southern Bolivia where sylvatic populations of *T. infestans* occur, the peri-domicile may represent not only the major source of insects that reinvade treated domiciles and peri-domicile complexes but also may act as a corridor between sylvatic and domestic populations. Vector movement is suggested by finding that *T. dimidiata* collected in and around houses had fed on opossum [26].

These methods have detection limits in terms of both taxonomic resolution, and quantity and quality of the blood meal. They are labor intensive [29], require considerable technical expertise, samples need to be fresh or frozen [30] and antibodies often are either not available commercially or the resources for synthesis and purification are not available. In a previous study we described an assay based on the polymerase chain reaction (PCR) amplification of a species-specific repetitive element of nuclear DNA to identify guinea pig DNA in the complex mixture present in the abdomen of *T. infestans*. A PCR based assay has advantages in that samples do not need to be fresh or frozen. The assay detects DNA quite soon after feeding, we detected guinea pig DNA in 100% of the vectors up to 40 hours after bugs had fed on guinea pigs under controlled conditions. In addition, starvation does not seem to have much impact on the ability to detect prior blood meals, in vectors collected from houses and peri-domestic areas of communities in the Department of Chuquisaca and maintained for two months without feeding, we were able to amplify guinea pig DNA in 8 of 34 samples demonstrating that at least 23% of the vectors had fed on guinea pigs [31].

In the present study we extend the assay to the identification of 11 vertebrate species, and the parasite *T. cruzi* using species-specific PCR assays. For cow, pig, chicken, dog, guinea pig, cat, mouse and donkey the assay targets short interspersed nuclear DNA elements (SINEs) [32]–[34]. The human assay detects the *Alu* element-based subfamily Yb6, a long interspersed element (LINE) [35]. Subunit 8 of the mitochondrial ATP synthase gene, *atp8* was amplified for goat and sheep [36]. We investigate three questions:

Are there differences among habitats (domestic vs peri-domestic) in the identity of the blood meals detected in the vectors? This information will give insight into vectors movement among habitats. For example, if domestic and peri-domestic vectors do not differ in the types of blood meals detected, that would indicate significant vector movement between habitats. At the other extreme, if human DNA is only detected in vectors collected in houses there is likely little movement from domestic to peri-domestic areas.

What is the frequency and pattern of single vs multiple blood meals? If vectors tend to feed on a single type of host, i.e., vectors feed on either dogs or humans but not both, then the likelihood of transmission among mammal species is reduced.

Does the rate of *Trypanosoma cruzi* infection vary among habitats and is it associated with specific blood meal types? Rates of *T. cruzi* infection in vectors will determine the probability of transmission to humans. High rates of *T. cruzi* infection in peri-domestic hosts enhance the risk for humans when insects migrate from peri-domestic to domestic habitats [15]. Because only mammals can sustain infection, *T. cruzi* infection should be low in bugs collected from chicken coops [27] if the bugs do not migrate and feed in other habitats. Finally, the likelihood of transmission varies among vertebrate hosts. Experiments have shown that bugs that feed on infected dogs are more likely to become infected than bugs feeding on infected humans [37].

## Methods

### Collection of vectors and description of study area

Eighty-one *T. infestans* collected from May to July of 2005 by Vector Control personnel at the Servicio Departamental de Salud (SEDES) of Chuquisaca, Bolivia were included in the present study. The insects were taken from a total sample of over one thousand insects collected from the department of Chuquisaca. The sample was stratified by habitat and insects were randomly taken from selected houses from each strata. Most insects were from domestic and peri- domestic sites in six northern localities in the department of Chuquisaca: 26 insects were collected in Capilla Llave (18°58′46S, 64°42′21″W), nine came from Carbajal (19°12′40″ S, 65°18′14″ W), 11 from Cueva Uyuni (19°25′0″S, 64°54′0″W), 13 from Serrano (19°06′0″S, 64°22′0″W) and four came from Yotala (19°9′31″S, 65°15′51″W). Eighteen insects from the southern locality of Huacareta (20°40′0″S, 63°45′0″W) were also examined.

Domiciles in this area have one or two adjacent bedrooms and a kitchen. A few have an additional room devoted to storing agricultural products; usually, part of a bedroom or a region of the kitchen is consigned to this purpose. The peri-domestic area includes structures such as pigpens; goat, sheep and cow corrals; and chicken coops. The animals, raised for subsistence, are usually at low density. Houses and fences of corrals are generally of adobe, combined with roofs of earth covered with split cane or ceramic tile. Thorn scrub branches are often also used in corrals atop adobe blocks to limit livestock movement and protect the adobe from rain. In the region under study, chickens live in a combined adobe-wall and wired enclosures with a thatched roof and they are not allowed to sleep within a room of the house, even during winter. All insects were collected from roof and walls of the enclosures.

Of the eighty-one *T. infestans* examined, 29 came from human habitations, 11 from chicken coops and 41 from corrals. There were 34 fifth, 12 fourth, and 11 third instar nymphs, one second and one first instars, 13 adult females and 10 adult males.

Upon collection, live insects were placed in plastic containers, transported to Sucre for species identification [38], placed in 96% ethanol, transported to Vermont, USA and stored at −20°C. No vertebrate animals were directly used in this study.

### Methods of molecular genetic analysis

DNA was extracted from ∼25 mg of tissue (cut from the posterior of the abdomen using a new razor blade for each individual to prevent contamination) using the DNeasy kit (Qiagen, Inc., Valencia, CA). DNA concentration was measured using a Nanodrop 1000 spectrophotometer (Nanodrop, Bethesda, MD).

Control DNA was extracted using the same kit. Positive controls for cow, pig, sheep and chicken, were from commercially purchased meat. Donkey, cat, dog and goat DNA were extracted from hair. Human DNA was from dried blood spots on filter paper. The blood was collected from Chuquisaca with the approval of the Ethics Board, University of San Francisco Xavier, Sucre, Bolivia. Mouse and guinea pig DNA were extracted from liver. The *T. cruzi* positive control was from previously sequenced samples that tested positive by PCR for *T. cruzi*.

Extracted DNA was amplified in 25 µl reactions using 1 µl of DNA template, 0.2 µM each of forward and reverse primers, 1× PCR Master Mix (0.2 mM dNTPs, 1.5 mM MgCl_2_, HotStart-IT *Taq* DNA polymerase and reaction buffer, USB Corporation, Cleveland, Ohio).

The PCR parameters for dog, guinea pig, cow, chicken, cat, mouse and pig DNA [33], [34] were: initial denaturation of 1 min at 95°C, followed by 30 cycles of 95°C for 30 s, annealing as shown in Table 1 for 30 s, and 30 s of extension at 72°C, except for the porcine assay where 1 minute to anneal and extend at 63°C was used.

**Table 1:** Primer sequence and PCR product size for species-specific assays.

Species		Primer A	Primer B	°C	size (bp)
Human^1^	*Homo sapiens*	tttgagacggagtctcgtt	gagatcgagaccacggtgaaa	61	200
Dog^2^	*Canis familiaris*	agggcgcgatcctggagac	agacacaggcagagggagaa	55	83
Guinea pig^2^	*Cavia porcellus*	gggatttagctcagtggcataag	attggtaccggggattgaact	60	71
Cow^2^	*Bos taurus*	tttcttgttatagcccaccacac	tttctctaaaggtggttggtcag	60	98
Chicken^2^	*Gallus gallus*	ctgggttgaaaaggaccacagt	gtgacgcactgaacaggttg	60	169
Cat^2^	*Felis catus*	agtcggttaagcgtctgacttt	ctccaggctctgagctgtca	55	98
Mouse^2^	*Mus musculus*	agatggctcagtgggtaaagg	gtggaggtcagaggacaaactt	55	118
Pig^2^	*Sus scrofa*	gactaggaaccatgaggttgcg	agcctacaccacagccacag	63	134
Donkey^2^	*Equus asinus*	ccaaagccccccagtacatag	gtggccaagtggttaagttcg	60	152
Sheep^3^	*Ovis aries*	ctcaaggagtattttgtttc	aattctatcaatattttttagt	48	117
Goat^3^	*Capra aegagrus*	tctcaaggggtgttatgc	gccacaactagacacatct	48	150
parasite^4^	*T. cruzi*	cgagctcttgcccacacgggtgct	cctccaagcagcggatagttcag	57	188

1 Nuclear Alu Yb6 subfamily [25].

2 Nuclear SINE (Short Interspersed Nuclear Element) [22], [23], [26].

3 Mitochondrial subunit 8 ATP synthase gene [24].

4 Nuclear 195 bp repetitive DNA [30].

For goat and sheep [36], the protocol was: 5 min at 95°C; 35 cycles of 92°C for 1 min, followed by annealing for 2 min at 48°C, extension for 2 min at 72°C; and final extension of 5 min at 72°C. For human [35] we used: 12 min at 95°C; followed by 40 cycles at 95°C for 15 s and 1 min at 61°C to anneal and extend. For donkey [32] the protocol was: initial denaturation for 5 min at 94°C; followed by 30 cycles of 94°C for 20 s, annealing for 30 s at 60°C, extension for 40 s at 72°C; and final extension of 6 min at 72°C. For *T. cruzi* [39], 10 min at 94°C was followed by 30 cycles of 94°C for 20 s, annealing for 10 s at 57°C, extension for 30 s at 72°C and a final extension of 7 min at 72°C. Amplicons were visualized by electrophoresis using 2% agarose, ethidium bromide staning and UV light. Positive and negative controls were always included.

Walker et al. [33], [34] describe an elegant series of assays demonstrating the specificity of the dog, pig, guinea pig, cow, chicken, cat and mouse primers for both single species and mixed species DNA samples. They also demonstrated the primers amplify DNA in quantities from 0.01 pg to 100 ng DNA/reaction depending on the species of target template. In a previous experimental study, we showed that the assay for guinea pig DNA was successful for 100% of the vectors up to 40 hours after bugs had fed. We were also able to detect guinea pig DNA in 8 of 34 field collected specimens maintained for two months without feeding prior to testing [31]. This is our first assay using the primers to detect multiple blood meals from vectors.

To rule out PCR inhibition by unknown components in the DNA extraction, we spiked the 12 samples in which no vertebrate or parasite DNA was detected with 0.2 ng of a mixture of *T. cruzi* and guinea pig DNAs and ran a second PCR. All of these showed amplification.

### Statistical methods

For most of the statistical analyses, the independent variables were categorical (habitat or species of vertebrate) and the dependent variable was ordinal (presence = 1 or absence = 0 for each vertebrate species or the *T. cruzi* parasite). For these analyses, we used contingency analysis combined with a likelihood ratio test (JMP 5.0.1.2, 2003). For statistical analysis that required another test, we describe that analysis below.

#### Variation among habitats in the types of blood meals detected

For each vertebrate host, we determined if each blood source was preferentially found in certain habitats. For example, was human DNA detected more often in domestic vectors than in peri-domestic vectors?

#### Frequency and pattern of multiple feeding

We tested whether insects that fed on a given host type were more or less likely to fed on other hosts. For example, did vectors in which we detected human DNA tend to feed exclusively on humans or did they test positive for more than one type of vertebrate host? We also determined if some pairs of DNA types co-occur in a single insect more or less often than predicted by random chance. Finally, the number of blood sources detected in a single vector was compared for domestic vs peri-domestic habitats using a nonparametric median test.

#### *Trypanosoma cruzi* association with habitats, vertebrate hosts and vector life stage

We examined if: (1) insects collected from domestic vs peri-domestic habitats differ in their likelihood of infection, (2) insects that had fed on different types of vertebrates varied in their likelihood of being infected with *T. cruzi* and (3) insects of different developmental stages differ in their likelihood of being infected.

## Results

We were able to identify a blood meal of at least one vertebrate species in 85% (69/81) of the vectors examined. Of the 12 samples in which no blood meal was detected, only one tested positive for *T. cruzi* DNA. A second aliquot of the negative samples spiked with a mixture of guinea pig and *T. cruzi* DNA showed the characteristic 188 bp and 71 bp bands for *T. cruzi* and guinea pig respectively, suggesting PCR inhibition was not a problem. None of the insects tested were positive for sheep, donkey, mouse or cat DNA. Pig blood was the most common type of blood meal, found in 34 of the 69 insects that tested positive for at least one type of vertebrate DNA (49%). Dog DNA was present in 24 cases (35%) and chicken in 18 (26%). All the other species were identified at a lower frequency (Table 2).

**Table 2:** Percent of vectors from each habitat that tested positive by PCR for 11 different types of vertebrate DNA.

Vertebrate host	N	Domestic	Peri-domestic	Likelihood ratio	p-value*
		%	%	Chi Square	
Human	13	41	2	22.14	<0.0001
Cow	9	17	8	1.65	n.s.
Chicken	18	7	31	7.06	<0.01
Pig	34	34	46	1.05	n.s.
Guinea pig	7	10	7	0.16	n.s.
Goat	11	7	17	1.89	n.s.
Dog	24	41	23	2.93	n.s.
Donkey, cat, sheep, or mouse	0	0	0		
No blood sources detected	12	10	17	0.75	n.s.

* Probability a blood source is randomly distributed between domestic and peri-domestic habitat types.

### Variation among habitats in the types of blood meals detected

Seven blood types were detected in insects collected from domestic habitats. Human and dog DNA were the most abundant in this ecotope, followed by pig cow, guinea pig, chicken and goat (Table 2). Of the 19 nymphs found in houses, DNA from non-domestic hosts was found in 14, while human, dog and/or guinea pig DNA was found in 15. Of the 7 adults collected from houses, 6 had DNA from non-domestic sources and 6 tested positive for human, dog and/or guinea pig DNA. In three domestic insects, no vertebrate blood was detected.

All species found in houses were also found in peri-domestic habitats. From the 42 insects collected from peri-domestic habitats, pig DNA was the most common, followed by chicken, dog, goat, guinea pig and human (Table 2). For nine peri-domestic, no vertebrate DNA was detected.

**Figure 1:**
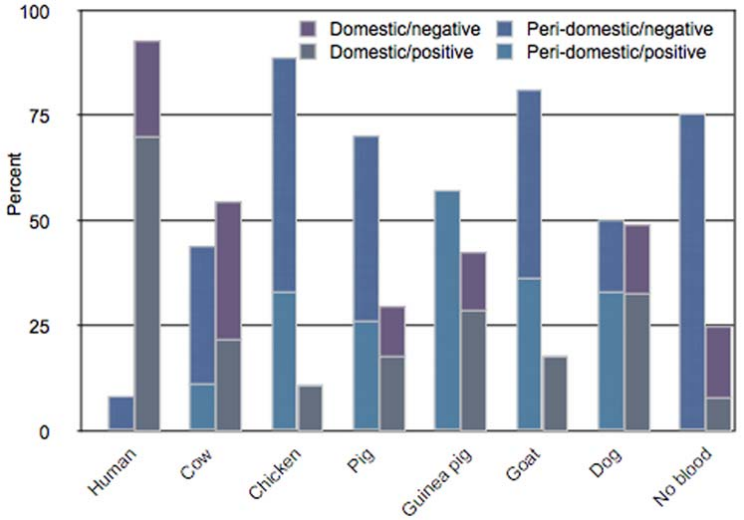
For domestic and peri-domestic habitats, we show the percentage of each type of blood meal found in that habitat and the *T. cruzi* prevalence in insect vectors that had fed on each blood source. Vectors from domestic vs. peri-domestic habitats vary in their blood meal source. Almost all the insects that had fed on humans were collected from houses. Detection of cow, chicken pig or goat DNA in vectors collected from houses indicates a migrant from a peri-domestic to domestic habitat. Detection of human DNA in a vector collected from a peri-domestic habitat indicates migration from domestic to peri-domestic habitat.

Human DNA is more prevalent in insects from houses than insects from peri-domestic habitats (Likelihood Ratio χ^2^ = 22.14, p<0.0001) and chicken DNA was more common in vectors from peri-domestic vectors than vectors from houses (Likelihood Ratio χ^2^ = 7.06, p<0.01), DNA of all the other vertebrate species is equally distributed between habitats (Figure 1 and Table 2). Those insects collected in houses that had fed on cow chicken pig or goat were defined as migrants, as were insects that had fed on humans and were found in corrals or chicken coops. Vectors are significantly more likely to migrate from houses to peri-domestic habitats than vise versa (Table 3).

**Table 3:** Vector movement between domestic and peri-domestic structures.

Habitat	N	migrant	non-migrant
Peri-domestic	41	1^1^	1
Domestic	23	15^2^	8

1 Number of vectors positive for human DNA collected from peri-domestic habitats.

2 Number of vectors positive for cow, chicken, pig or goat DNA collected in houses.

3 Likelihood Chi-square = 32.86, p<0.0001).

### Frequency and pattern of multiple feeding

From the 69 samples that tested positive for at least one type of vertebrate or *T. cruzi* DNA, we detected one blood meal in 36 (52%) insects. For 22 (32%) specimens we were able to detect two different vertebrate species, in 8 we detected three different types of vertebrate DNA and finally in 3 insects we were able to identify the presence of four different types of vertebrate DNA (Table 4). The mean number of distinct blood sources detected in a single insect was significantly different among chicken coops (1.64), bedrooms (1.59), and corrals (1.27) (Median Test: Chi-square = 9.26, d.f. = 2, p<0.01). The likelihood of finding a particular blood meal mixed with at least one other type of blood (Table 4). In contrast, it was more likely that there was only one source when bugs fed on humans or cows. Pairwise co-occurrence and significant associations between species when two types of DNA were detected in a single bug are shown in Table 5. When insects were found to have fed on pigs, the frequency of human blood was significantly less than expected by chance (p<0.05). This was also the case between pig and goat DNA when both types of DNA were found in the same sample. When dog DNA was detected the probability of guinea pig DNA in the same insect was higher than expected (p<0.05).

**Table 4:** Frequency of single vs multiple (2, 3 or 4) vertebrate blood types identified by PCR in T. infestans from Chuquisaca, Bolivia.

	Single	2	3	4	Likelihood ratio Chi Square	p-value*
Human	5	5	2	1	2.73	n.s.
Cow	2	5	2	0	5.78	<0.05
Chicken	4	10	3	1	13.29	<0.001
Pig	16	9	6	3	3.61	<0.05
Guinea Pig	1	1	3	2	6.64	<0.05
Goat	5	3	1	2	0.99	n.s.
Dog	3	11	7	3	32.74	<0.0001
Total**	36	22	8	3		

* Probability that vectors that fed on the specified host fed on more than one host (one-tailed test).

** Total number is less than the column sum because of multiple infections.

**Table 5:** Pairwise probabilities of occurrence of two types of DNA in the same T. infestans specimen from Chuquisaca.

	Human	Cow	Chicken	Pig	Guinea Pig	Goat
Cow	0.27*					
	n.s.					
Chicken	0.45	0.00				
	n.s.	n.s.				
Pig	5.00	0.32	0.09			
	(<0.05) ⇓	n.s.	n.s.			
Guinea Pig	2.57	1.73	3.70	0.71		
	n.s.	n.s.	n.s.	n.s.		
Goat	0.52	0.05	1.50	3.23	4.10	
	n.s.	n.s.	n.s.	n.s.	(<0.05) ⇑	
Dog	0.56	1.96	0.92	0.90	10.60	0.03
	n.s.	n.s.	n.s.	n.s.	(<0.01) ⇑	n.s.

* Likelihood ratio Chi square (P-values) for pair co-occurring less than (⇓) or greater than (⇑) expected by chance.

### *Trypanosoma cruzi* association with habitats, vertebrate hosts and vector life stage

Infection with *T. cruzi* was detected in 34 out of 81 bugs (42%). The prevalence of infection was higher in insects from houses (55%) compared to that observed in chicken coops (27%) and corrals (37%); however, these differences are not statistically significant (Table 6). Insects that had fed on humans, dogs or guinea pigs were more likely to be infected by *T. cruzi* (p<0.05) (Table 7, Figure 1). No significant difference in the frequency of *T. cruzi* infection was observed by developmental stage of the insect (X^2^ = 10.44, p>0.05) data not shown.

**Table 6:** Variation among habitats in T. cruzi prevalence in T. infestans from Chuquisaca.

PCR result	Sample size	Domicile	Chicken coop	Corral
Negative	47	13	8	26
Positive	34	16	3	15
% Positive*	42%	55%	27%	37%

* T. cruzi infection prevalence not significantly different among habitats (Likelihood ratio Chi square = 3.56, P>0.05).

**Table 7:** T. cruzi association with vertebrate hosts of T. infestans from Chuquisaca.

Vertebrate host	Likelihood ratio Chi square*	P value
Human	4.70	<0.05⇑
Cow	0.32	n.s.
Chicken	0.06	n.s.
Pig	0.11	n.s.
Guinea pig	6.29	<0.05⇑
Goat	0.82	n.s.

* Likelihood ratio Chi square (P-values) for pair co-occurring less than (⇓) or greater than (⇑) expected by chance.

## Discussion

Reduction of the incidence of Chagas disease requires knowledge of how vectors move among habitats and especially the source of vectors that colonize houses after insecticide application. Our results show that this method can be used to show movement from peri-domestic structures to human habitations for *T. infestans* as well as the important role of pigs, dogs, chickens and guinea pigs in the dynamics of *T. cruzi* infection. Spatial analysis of variation among habitats demonstrates: (1) significantly more movement from peri-domestic to domestic structures than vice versa and (2) that vectors testing positive for human and chicken DNA are more likely to be collected from domestic structures and peri-domestic structures, respectively. Vectors testing positive for the other types of vertebrate DNA appear to be randomly distributed among habitats. Almost half of the vectors tested positive for more than one vertebrate species, *T. cruzi* infection was detected in 42% of the specimens and there was a significant difference in *T. cruzi* infection between domestic and peri-domestic habitats.

The method was used to show a dispersal from peri-domestic habitats toward the domicile. Vectors that had fed on humans were predominantly collected in human habitations, and only one insect that had fed on humans was collected outside of houses. In contrast, blood of six species of peri-domestic hosts was found in domestic vectors. Dogs and pigs provided the majority of the blood meals detected in the peri-domicile; and in vectors collected in houses, these hosts were the most common after humans. The likelihood of hosts spatially co-occurring affects the likelihood of finding vectors that have fed on both: pig blood was negatively associated with human blood, and guinea pig blood was positively associated with dog and goat blood.

The results of a study in rural villages of north-west Argentina showed *T. infestans* feeding patterns are non-random, the likelihood of feeding on humans decreased when dogs and chickens were present in bedroom areas [20]. The role of pig corrals as reservoirs has also been reported from studies in Argentina [28], [40]. Our insect collection methods did not include counting potential vertebrate hosts in the domicile or peri-domicile; however we do note that cow DNA was found more frequently than goat DNA in insects collected in houses. Host abundance and proximity are often considered the main determinants of host choice [20]. In this region, cows are usually kept in open corrals adjacent to human dwellings while goats are often enclosed in more distant corrals constructed with adobe blocks. The number of cows per family is usually lower than the number of goats.

We have found a high rate of *T. cruzi* infection in insects collected in houses (55%) and a significantly higher rate of *T. cruzi* infection in bugs that had fed on humans, dogs and guinea pigs. These data contrast markedly with the 4.6% of infected insects found during the surveillance phase in northwestern Argentina [42] and is consistent with the rate of 58% reported for *sylvatic T. infestans* in the Andean Valleys of Bolivia [43], [44] and the current high levels of transmission in this part of Bolivia. The high prevalence of *T. cruzi* in insects collected from houses could partially be due to the close proximity of dogs and guinea pigs, which also maintain a high parasite burden. Dogs are often employed as animal guards, watching livestock during the day and returning to the house in the evening. In this movement, they may transport *T. cruzi* from one habitat to another. Although, dogs in this area are not usually allowed to enter houses, they often sleep outside against the bedroom wall. Rural families in this part of Bolivia maintain guinea pigs for food in corrals located close to the house or in a particular compartment in the kitchen. We found four insects in corrals that had fed on guinea pigs. As wild guinea pigs occur in burrows in stone fences of goat and pig corrals (personal observation) in this area, we cannot ascertain whether these blood meals were from domestic or sylvatic animals.

The use of forensic DNA analysis to examine feeding in disease vectors [45]–[50], including vectors of Chagas disease is relatively new [51]. Our use of primers based on SINEs and LINEs that have high copy number and small size is advantageous for degraded DNA. For nine of the eleven vertebrate taxa we investigate, the assay is based on highly repetitive, relatively small (∼70–200 bp) sequences of Large or Small Interspersed Nuclear Element (LINE or SINE) transposable DNA, which can be an advantage when using degraded DNA. A negative PCR result for blood meal, observed in 12 specimens, could be because the insect had not fed, failure of PCR, or the blood meal source was a species not included in our assay. In 11 of these 12 samples we did not find evidence of *T. cruzi* infection. The *T. cruzi* found in the blood meal negative bug could have also been a result of co-prophagy. One specimen that tested negative for all 11 vertebrate species was a first instar nymph and could be unfed. Experimental work showed our assay detects DNA in 100% of specimens (N = 36) in as little as 1 hour and up to 40 hours after controlled feeding and identified guinea pig DNA in 23% (9 of 34) of *T. infestans* collected in the wild and maintained for two months, without feeding, under controlled conditions in the laboratory [31]. Estimates of mean feeding intervals in field-collected *T. infestans* from Argentina ranged from 3 to 7 days [41]. Thus, it is unlikely that our negative results for the other specimens were because insects were unfed. Insufficient DNA in the extracted sample or degraded DNA may offer an alternative explanation for the negative results since some specimens were analyzed even eight months after collection. In a previous study using these primers, the detection limits in complex (mixed) DNA samples using 2% agarose gels and ethidium bromide are: 5 pg in a 10 ng mixture (0.05%) for chicken, 0.005% for cow and 0.0005% for pork [33]. Non-reactive results ranged from 7% to 14% in a study on feeding patterns in *T. infestans* determined by double-diffusion using five genus-specific antisera [41] and 14% of *Triatoma sp.*specimens showed no amplification when tested for vertebrate cytB blood feedling [51]. PCR inhibition in our assay can be ruled out since negative samples amplified the two species DNA after “spiking” an aliquot of each sample with a mixture *T. cruzi* and guinea pig DNA's. We looked for 11 different domestic and peri-domestic species; however, we cannot eliminate the possibility that insects had fed on other species.

Although we detected guinea pig DNA in 100% of *Triatoma infestans* up to 40 hours after experimental feeding, a study of blood-feeding in two species of mosquitoes (*Anopheles stephensi* and *Culex quinquefasiatus*) using PCR amplification of a 358 bp region of the mtDNA cytB gene reported that all mosquitoes tested positive 1 and 6 hours after feeding, but at starting at 12 hours, there was a negative relationship between time since feeding and success of PCR amplification. There was no difference in the ability to detect blood meals between the two mosquito species, or between mosquitoes stored at +4 or −20°C up to 30 h after meal ingestion [49]. The results of an experiment of in *Triatoma pallidipennis* feeding on BALB/c mice amplified a 420 bp region of cytB DNA [51]. Host DNA could be detected at least until 10 weeks after the blood meal; however not all bugs tested positive for mouse DNA. Bugs that had fed for <5 min produced less intense bands or no amplification. Mota et al. [51] also reported that DNA obtained from insects collected and preserved for as long as 6 years in 70% ethanol could be amplified.

In conclusion, we have evaluated PCR-based assays for detection of the blood meal source in *T. infestans*. These assays tested for 11 species and were able to detect up to four species of DNA from a complex mixture in the abdominal content of bugs. Most of the species are detected based on small sized amplicons of nuclear DNA sequences with a high copy number, making them more likely to work with small amounts or degraded DNA. In addition, the expertise and equipment to perform these assays are amenable to medium equipped laboratories that may be available in many Chagas disease endemic regions.

From the epidemiological point of view, this study has disclosed the important role that pigs, dogs, chickens and guinea pigs exercise in the dynamics of the *T. cruzi* infection as well as in the regulation of dispersal patterns of *T. infestans* in this region of Bolivia.
